# Supplementary material for: Olfactory Learning in the Stingless Bee Melipona eburnea Friese (Apidae: Meliponini)
Source: Insects. 2019 Nov 18;10(11):412. doi: 10.3390/insects10110412 (PMC6920981; doi:10.3390/insects10110412)
Supplement: Supplementary file 1 [file insects-10-00412-s001.pdf]

Supplementary Material 1

Olfactory Learning in the Stingless Bee *Melipona eburnea* Friese

Marisol Amaya-Márquez*, Sergio Tusso, Juan Hernández, Juan Darío Jiménez, Harrington Wells, Charles I. Abramson

*Correspondence: mamayam@unal.edu.co

Olfactory conditioning of harnessed bees using the proboscis extension reflex (PER)

We evaluated absolute and differential scent conditioning of *Melipona eburnea*. The terms used here, follow previous definitions of conditioning [49]. The study was conducted June and July 2013 in the Cordillera Oriental of the Colombian Andes, Department of Cundinamarca, municipality of Fusagasuga. The *Melipon*a apiary was located in the village of Bosachoque. For each experiment, workers were collected from the hive entrance as they left for foraging using a wire frame mesh cylinder. Without chilling the bees, each bee was harnessed in a 9 mm ID plastic tube 20 mm long. We offered the bees water before placing them in darkness at room temperature for 1 hr prior to an experiment.

Sucrose Response Threshold (SRT): Sugar Response Threshold (SRT) is the minimal sugar concentration at which the PER response occurs, and is basic in PER conditioning studies [50]. SRT is useful to know the range and the optimal sugar concentration to which species, colonies, or individuals respond. Here we evaluate SRT to determine the sucrose solution to which the stingless bee *M. eburnea* respond better and use that value in the conditioning procedure. We followed the SRT methodologies of [34,50] with few modifications. The series of sucrose solutions 0.1, 0.3, 1.0, 3.0, 10.0, 30.0 and 50.0% (w/w) was presented to each bee by touching the antenna with different toothpicks soaked in the different sucrose solutions. Each bee (n = 142 bees) was given each sucrose concentration once. Sucrose solutions were given in order of increasing sucrose concentration. At the beginning and between sucrose trials the bees were offered water to avoid confounding effects of thirst, and to control for sensitization and habituation [34]. The PER response was recorded only when the bee extended its proboscis completely.

Absolute olfactory conditioning: We evaluated classical PER conditioning of *M. eburnea* to scent following the PER protocol used with the honey bee, *Apis mellifera* [13, 33]. Only bees upon capture that 1) showed the PER unconditioned response (UR) of extending their proboscis when the antenna was contacted with the sucrose solution, and 2) did not show PER spontaneous response to any of the scents we used in the experiment.

Either cinnamon or lavender scent (Lorann oil) was used as the conditioned stimulus (CS) and a 50% (w/v) sucrose solution was used as the unconditioned stimulus (US). Half the bees received the cinnamon CS and the other half the lavender CS (total of n=30 bees). The experiment consisted of 12 trials per bee. The inter trial interval (ITI) was 10 minutes. The experiment used a non-overlapping CS–US design. Trials were 7 sec, 5 sec of CS airflow followed by presentation of the US for 2 sec. We used a syringe to deliver the airflow [5, 44] with a 1 cm square piece of Whatman no.1 filter paper attached to the syringe-plunger tip. A different syringe was used for each scent. The 5 sec CS had 4 μL of the scent essence placed on the filter paper.

Differential olfactory conditioning: We evaluated differential PER conditioning to scent following previous methodologies [34, 37]. Like the prior experiment, only bees upon capture that, 1) showed the PER unconditioned response, and 2) did not show PER spontaneous response to the scents, were used in the experiment (n = 160 bees, 40 per test group).

Clove and strawberry scents (Lorann oils) were used as the conditioned stimulus (CS) and a 50% (w/v) sucrose solution as the unconditioned stimulus (US). The CS+ was paired with the US while the CS– was not paired with the US. The four test groups were: 1) strawberry CS+ with clove CS–, and 2) clove CS+ with strawberry CS–. 3) cinnamon CS+ with strawberry CS–, and 4) clove CS+ with cinnamon CS–. The experiment used 12 pseudorandom trials per bee (6 CS+ and 6 CS–). The inter trial interval (ITI) used was 10 minutes. Both CS+ and CS– trials lasted 6 sec: 6 sec of CS airflow. For the CS+ trials an overlapping design was used with the last 3 sec of CS airflow overlapped with the delivery of the US. The delivery of the CS was the same as for the prior experiment (n= 160 bees, 40 per test group).

As a positive control we followed the same protocol but used Africanized honey bees (*Apis mellifera* var. *scutellata*). We used strawberry CS+ with clove CS– (n= 40 bees).

Results

Sucrose Response Threshold (SRT): The stingless bees *M. eburnea* showed a wide range of sucrose threshold response (STR) values, with some bees eliciting the PER to sucrose solutions 0.1% w/w while in other bees only sucrose solutions 50% w/v elicited PER. Most bees showed a SRT to 30% w/w sucrose solutions (Figure S1).

Absolute olfactory conditioning: The stingless bee *M. eburnea* did not show olfactory learning using the harnessed-bee PER conditioning protocol. The total lack of response to the CS scent conditioning occurred in both tests conducted: one with cinnamon CS and the other with lavender CS (Figure S2).

Differential olfactory conditioning: The stingless bee *M. eburnea* did not show olfactory learning in any of the four differential conditioning tests conducted using the PER protocol with harnessed bees (n = 160). In fact, none of the bees responded even once to either the CS+ or CS- (Fig. S3). However, the use of the PER protocol with the Africanized honey bee *A. mellifera scutellata* as a positive control was satisfactorily demonstrated (Figure S4). The bees responded to the (CS+) at the second trial and by the six trial 70 % of the tested bees (n = 40) respond only to the CS+.

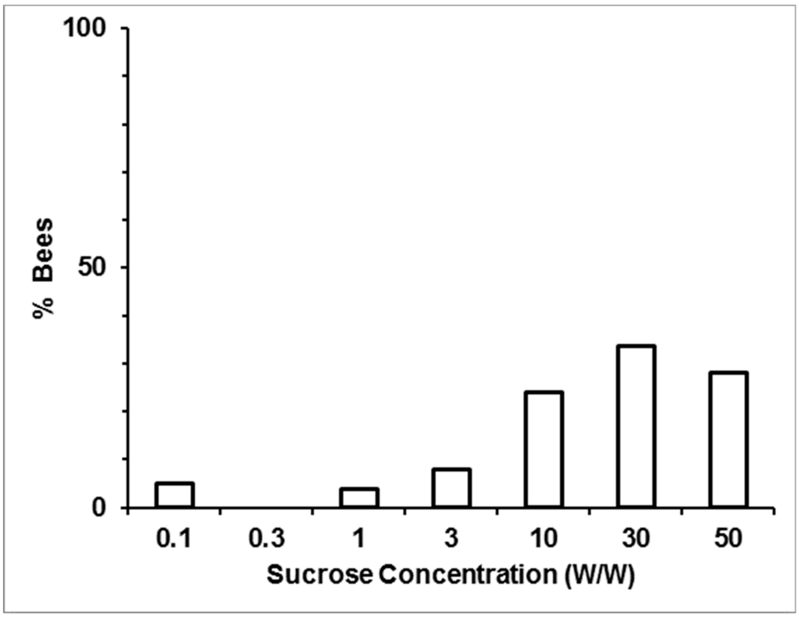

Figure S1. Sucrose Response Threshold (SRT) eliciting the Proboscis Extension Reflex (PER) in the stingless bees *Melipona eburnea*.

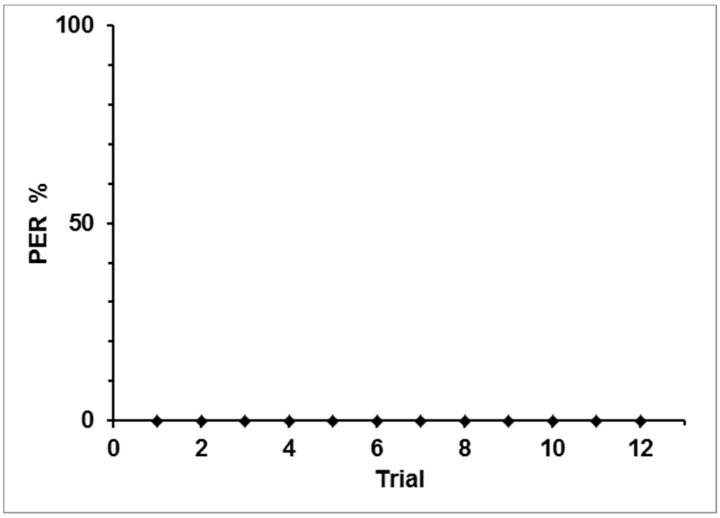

Figure S2. Absolute conditioning using harnessed stingless bees, *Melipona eburnea.* Both the results for lavender as the CS and for cinnamon as the CS are displayed at each trial. None of the bees responded to either CS.

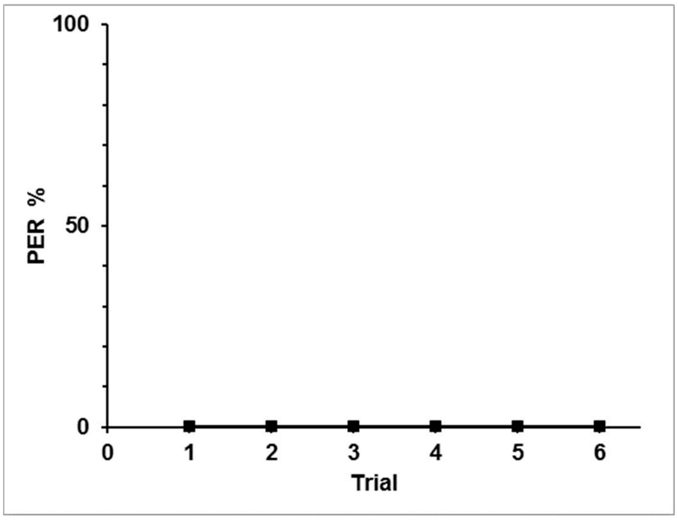

Figure S3. Differential scent conditioning to PER (Proboscis Extension Reflex) using harnessed stingless bees, *Melipona eburnea*. All four differential conditioning groups (CS+/CS- odor pairs) are shown, and the percentage of conditioned PER responses both to the CS+ and CS– are shown in each case. None of the bees responded to either the CS+ or CS–.

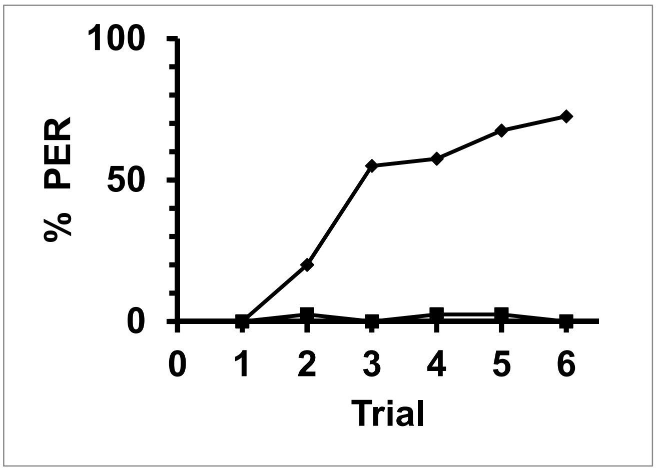

Figure S4. Experimental Positive Control: differential scent conditioning to PER (Proboscis Extension Reflex) using the Africanized honey bee *Apis mellifera* var. *scutellata*. Percentage of bees showing the Proboscis Extension Reflex (PER) to the (CS+) ♦ and (CS–) ■ over 12 training trials (6 CS+ and 6 CS– presented in a pseudo-random order).

2.2. Absolute conditioning of free-moving (fm) bees using the proboscis extension reflex PER (fmPER)

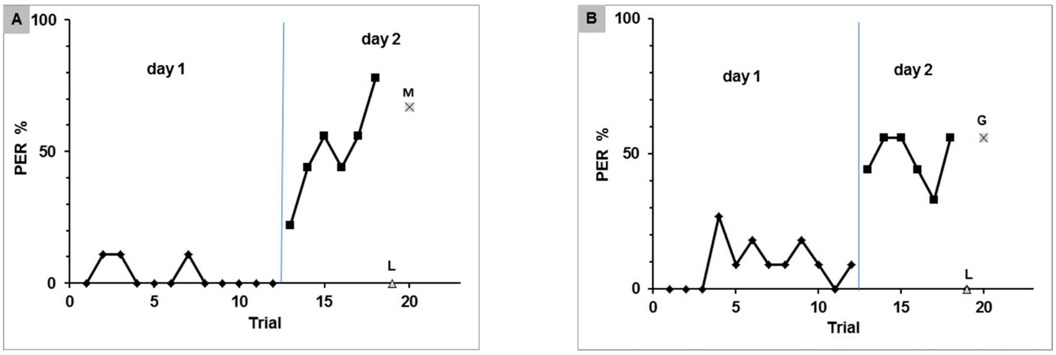

Figure S5. Experiments 1 (A) and 2 (B). **CS+ same scent days 1 and 2, US unscented** using free-moving stingless bees (fmPER) *Melipona eburnea*. **A**. Mango CS. **B.** CS Guava CS. PER response to the novel scent and to the CS+ conditioned on days 1 and 2 is depicted at the end of the training. First trial of day 2 (= trial 13). (L): lavender, (G): guava, (M): mango.

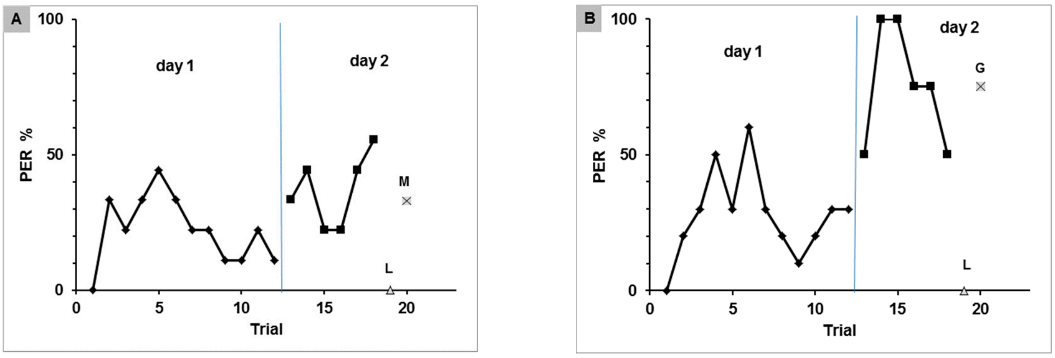

Figure S6. Experiments 3 (A) and 4 (B). **CS+ same scent days 1 and 2, US scented** with same scent as CS+ using free-moving stingless bees (fmPER) *Melipona eburnea.* **A**. Mango CS & US. **B**. Guava CS & US. PER response to the novel scent and to the CS+ conditioned on days 1 and 2 is depicted at the end of the training. First trial of day 2 (= trial 13). (L): lavender, (G): guava, (M): mango.

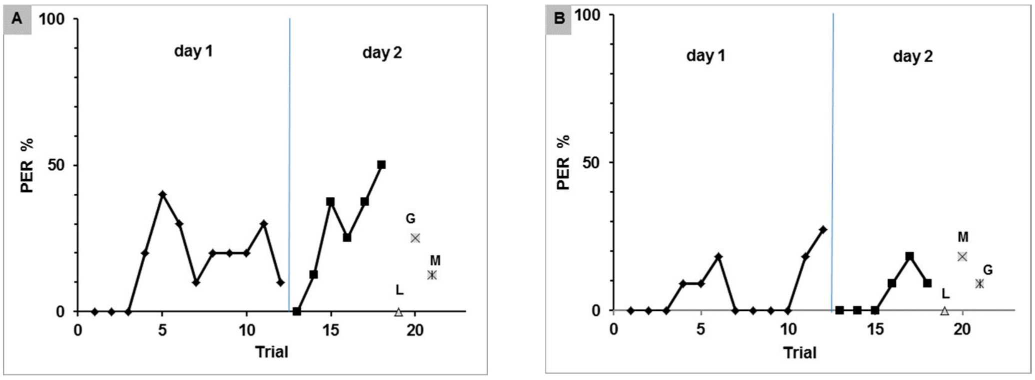

Figure S7. Experiments 5 (A) and 6 (B). **CS+ different scent days 1 and 2, US unscented**, using free-moving stingless bees (fmPER) *Melipona eburnea* **A**. Mango day 1, Guava day 2. **B**. Guava day 1, Mango day 2. PER response to the novel scent and to the CS+ conditioned on days 1 and the CS+ conditioned on day 2 is depicted at the end of the training. First trial of day 2 (= trial 13). (L): lavender, (G): guava, (M): mango.

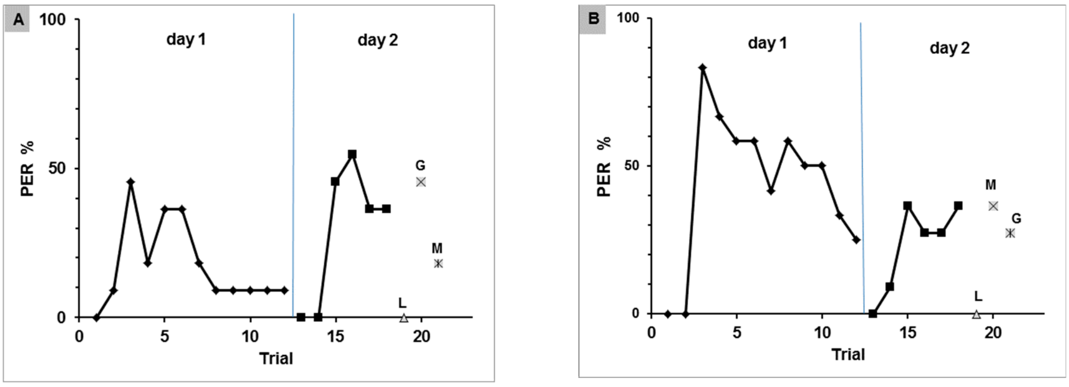

Figure S8. Experiments 7 (A) and 8 (B). **CS+ different scent days 1 and 2, US scented** with the scent as CS+ each day, using free-moving stingless bees (fmPER) *Melipona eburnea.* **A**. Mango day 1, Guava day2. **B**. Guava day 1, Mango day 2. PER response to the novel scent and to the CS+ conditioned on days 1 and the CS+ conditioned on day 2 is depicted at the end of the training. First trial of day 2 (= trial 13). (L): lavender, (G): guava, (M): mango.

Supplementary Material 2

Olfactory Learning in the Stingless Bee *Melipona eburnea* Friese

Marisol Amaya-Márquez*, Sergio Tusso, Juan Hernández, Juan Darío Jiménez, Harrington Wells, Charles I. Abramson

*Correspondence: mamayam@unal.edu.co

Table S1: Selection Model day 1, based on the information indexes AIC and BIC.

Model		Df	AIC	BIC	P-value (>Chisq)
MODEL_DAY1_5	CONDITIONED_RESPONSE ~ TRIALS + REWARD + ODOR + (1 | BEE_TOTAL)	5	379.30	400.35	
MODEL_DAY1_4	CONDITIONED_RESPONSE ~ TRIALS + REWARD + ODOR +TRIALS:ODOR + (1 | BEE_TOTAL)	6	379.89	405.15	0.23465
MODEL_DAY1_3	CONDITIONED_RESPONSE ~ TRIALS + REWARD + ODOR + TRIALS:REWARD + TRIALS:ODOR + (1 | BEE_TOTAL)	7	380.20	409.67	0.19348
MODEL_DAY1_2	CONDITIONED_RESPONSE ~ TRIALS + REWARD + ODOR + TRIALS:REWARD + TRIALS:ODOR + REWARD:ODOR + (1 | BEE_TOTAL)	8	381.97	415.65	0.63157
MODEL_DAY1_1	CONDITIONED_RESPONSE ~ TRIALS * REWARD * ODOR + (1 | BEE_TOTAL)	9	380.54	418.44	0.06428 .

Table S2: Selection Model day 2, based on the information indexes AIC and BIC.

Model		Df	AIC	BIC	P-value (>Chisq)
MODEL_DAY2_12	CONDITIONED_RESPONSE ~ TRIALS + EXPERIENCE + ODOR + TRIALS:EXPERIENCE+ (1 | BEE_TOTAL)	6	378.00	402.41	
MODEL_DAY2_11	CONDITIONED_RESPONSE ~ TRIALS + EXPERIENCE + REWARD + ODOR + TRIALS:EXPERIENCE + (1 | BEE_TOTAL)	7	379.21	407.69	0.37566
MODEL_DAY2_10	CONDITIONED_RESPONSE ~ TRIALS + EXPERIENCE + REWARD + ODOR +TRIALS:EXPERIENCE + EXPERIENCE:REWARD ODOR + (1 | BEE_TOTAL)	8	380.56	413.11	0.41884
MODEL_DAY2_9	CONDITIONED_RESPONSE ~ TRIALS + EXPERIENCE + REWARD + ODOR + TRIALS:EXPERIENCE + EXPERIENCE:REWARD + EXPERIENCE:ODOR+ (1 | BEE_TOTAL)	9	382.23	418.85	0.56550
MODEL_DAY2_8	CONDITIONED_RESPONSE ~ TRIALS + EXPERIENCE + REWARD + ODOR + TRIALS:EXPERIENCE + EXPERIENCE:REWARD + EXPERIENCE:ODOR + REWARD:ODOR+ (1 | BEE_TOTAL)	10	384.19	424.87	0.83747
MODEL_DAY2_7	CONDITIONED_RESPONSE ~ TRIALS + EXPERIENCE + REWARD + ODOR + TRIALS:EXPERIENCE + EXPERIENCE:REWARD + EXPERIENCE:ODOR + REWARD:ODOR + EXPERIENCE:REWARD:ODOR + (1 | BEE_TOTAL)	11	382.71	427.46	0.06205
MODEL_DAY2_6	CONDITIONED_RESPONSE ~ TRIALS + EXPERIENCE + REWARD + ODOR + TRIALS:EXPERIENCE + EXPERIENCE:REWARD + TRIALS:ODOR + EXPERIENCE:ODOR + REWARD:ODOR +EXPERIENCE:REWARD:ODOR+(1 | BEE_TOTAL)	12	381.28	430.10	0.06398
MODEL_DAY2_5	CONDITIONED_RESPONSE ~ TRIALS + EXPERIENCE + REWARD + ODOR + TRIALS:EXPERIENCE + EXPERIENCE:REWARD +TRIALS:ODOR + EXPERIENCE:ODOR + REWARD:ODOR + TRIALS:EXPERIENCE:ODOR + EXPERIENCE:REWARD:ODOR+ (1 | BEE_TOTAL)	13	381.38	434.27	0.16885
MODEL_DAY2_4	CONDITIONED_RESPONSE ~ TRIALS + EXPERIENCE + REWARD + ODOR+TRIALS:EXPERIENCE + TRIALS:REWARD +EXPERIENCE:REWARD +TRIALS:ODOR + EXPERIENCE:ODOR + REWARD:ODOR + TRIALS:EXPERIENCE:ODOR + EXPERIENCE:REWARD:ODOR+ (1 | BEE_TOTAL)	14	382.74	439.70	0.42245
MODEL_DAY2_3	CONDITIONED_RESPONSE ~ TRIALS + EXPERIENCE + REWARD + ODOR + TRIALS:EXPERIENCE + TRIALS:REWARD + EXPERIENCE:REWARD + TRIALS:ODOR + EXPERIENCE:ODOR + REWARD:ODOR + TRIALS:EXPERIENCE:REWARD + TRIALS:EXPERIENCE:ODOR + EXPERIENCE:REWARD:ODOR+(1 | BEE_TOTAL)	15	384.55	445.58	0.66509
MODEL_DAY2_2	CONDITIONED_RESPONSE ~ TRIALS + EXPERIENCE + REWARD + ODOR TRIALS:EXPERIENCE + TRIALS:REWARD + EXPERIENCE:REWARD + TRIALS:ODOR + EXPERIENCE:ODOR + REWARD:ODOR + TRIALS:EXPERIENCE:REWARD + TRIALS:EXPERIENCE:ODOR + TRIALS:RE	16	386.53	451.62	0.88110

	WARD:ODOR + EXPERIENCE:REWARD:ODOR +<br>(1 | BEE_TOTAL)				
MODEL_DAY2_1	CONDITIONED_RESPONSE ~ TRIALS * EXPERIENCE * REWARD * ODOR + (1 | BEE_TOTAL)	17	388.51	457.67	0.87658
